# *In-situ* Effects of Eutrophication and Overfishing on Physiology and Bacterial Diversity of the Red Sea Coral *Acropora hemprichii*

**DOI:** 10.1371/journal.pone.0062091

**Published:** 2013-04-22

**Authors:** Christian Jessen, Javier Felipe Villa Lizcano, Till Bayer, Cornelia Roder, Manuel Aranda, Christian Wild, Christian R Voolstra

**Affiliations:** 1 Coral Reef Ecology Group (CORE), Leibniz Center for Tropical Marine Ecology (ZMT), Bremen, Germany; 2 Red Sea Research Center, KAUST, Thuwal, Saudi Arabia; 3 Faculty of Biology and Chemistry, University of Bremen, Bremen, Germany; Argonne National Laboratory, United States of America

## Abstract

Coral reefs of the Central Red Sea display a high degree of endemism, and are increasingly threatened by anthropogenic effects due to intense local coastal development measures. Overfishing and eutrophication are among the most significant local pressures on these reefs, but there is no information available about their potential effects on the associated microbial community. Therefore, we compared holobiont physiology and 16S-based bacterial communities of tissue and mucus of the hard coral *Acropora hemprichii* after 1 and 16 weeks of *in-situ* inorganic nutrient enrichment (via fertilizer diffusion) and/or herbivore exclusion (via caging) in an offshore reef of the Central Red Sea. Simulated eutrophication and/or overfishing treatments did not affect coral physiology with respect to coral respiration rates, chlorophyll *a* content, zooxanthellae abundance, or δ ^15^N isotopic signatures. The bacterial community of *A. hemprichii* was rich and uneven, and diversity increased over time in all treatments. While distinct bacterial species were identified as a consequence of eutrophication, overfishing, or both, two bacterial species that could be classified to the genus *Endozoicomonas* were consistently abundant and constituted two thirds of bacteria in the coral. Several nitrogen-fixing and denitrifying bacteria were found in the coral specimens that were exposed to experimentally increased nutrients. However, no particular bacterial species was consistently associated with the coral under a given treatment and the single effects of manipulated eutrophication and overfishing could not predict the combined effect. Our data underlines the importance of conducting field studies in a holobiont framework, taking both, physiological and molecular measures into account.

## Introduction

Coral reefs are threatened by global (e.g. ocean acidification, ocean warming) and local (e.g. coral disease outbreaks, overfishing, eutrophication) anthropogenic stressors [1]–[4], whereby eutrophication and overfishing are among the two most important local factors affecting coral reef health [3]. On a macro-ecological scale, overfishing can reduce resilience of hard corals and the reefs that they are engineering [5] in numerous ways. Reduced herbivore stocks can lead to prolonged recovery times of corals after disturbances [6], increase crown-of-thorns starfish outbreaks [7], and release macroalgae from their top-down control [3]. Eutrophication, and more specifically nutrient enrichment, can have severe direct effects on scleractinian corals by hampering coral reproduction [8], reducing calcification [9], [10], reducing the threshold of heat- and light stress mediated bleaching [11], advancing coral disease [12], and shifting microbial communities towards bacteria associated with diseased corals [13]. On the other hand, eutrophication affects scleractinian corals also indirectly by increasing turf and macroalgae [14]–[17], inhibit coral recruitment [18], [19], or directly outcompete corals via allelochemicals [20]–[22]. Furthermore, turf and macroalgae increase microbial activity and trigger changes in the microbial community associated with corals [23]–[26] by dissolved organic carbon (DOC) release that can accelerate microbial growth and create a positive feedback loop when corals die and free space for more algae [27], [28].

The basic functional unit of coral reefs is the coral holobiont [29] that consists of a complex symbiotic interaction between the coral animal, its intracellular photosynthetic algae, and a wide spectrum of extra- and intracellular bacteria, archaea, fungi, eukaryotes, and viruses [29]–[32]. In contrast to the long history of studying the coral-algal relationship, understanding the importance of the bacterial assemblage in corals has only been recently targeted. Initial studies showed that the microbial community of corals is diverse, complex, and uneven [31]–[33]. Furthermore, bacterial communities are species-specific [31], [33] and differ on a spatial [32], [34], as well as a temporal scale [35]. Unlike most zooxanthellae, bacteria seem to be both stochastically and horizontally transmitted to the coral [31], [36]. Their establishment, however, may depend on the variables associated with the environment and their genetic capability to colonize the coral niche under those specific conditions [37]. It becomes clear that corals are meta-organisms and their phenotypic responses are a result of the complex interplays between all member species. Hence, in order to comprehensively understand coral physiology, it is important to look at the interactions between host and symbionts and the prevailing environmental conditions.

A number of previous studies investigated the bacterial diversity in coral reef organisms via 16S amplicon sequencing [32], [34], [35], [38]–[40]. However, many of these studies analyzed a single point in time and space with limited indications towards the variance and stability of the microbial assemblage of the coral holobiont. Given the rate of decline of coral reef cover worldwide [41], it is becoming increasingly important to understand coral reef and coral holobiont functioning by combining physiological and molecular measures. Despite the fact that coral reefs in the Red Sea are of relative pristine condition, they are, too, challenged by the effects of coastal development and overfishing [42]. Hence, we set out to analyze coral physiology and associated changes of the microbial community of *Acropora hemprichii* in the Central Red Sea after 1 and 16 weeks of *in situ* artificial nutrient enrichment (using a fertilizer with 15% nitrogen, 9% phosphate, 12% potassium oxide, and trace metals), *in situ* herbivore exclusion (using cages with a mesh size of 4 cm to exclude larger herbivores), and a combined treatment to simulate effects of eutrophication and overfishing. While the latter two processes generally refer to ecosystem scale effects, we were investigating effects on the scale of the holobiont in this study. To our knowledge, this is the first study from the Red Sea that evaluates top-down (i.e. herbivory) and bottom-up (i.e. nutrient availability) factors on coral holobiont functioning and provides first insights into the effects on coral physiology and associated bacterial community changes.

## Materials and Methods

### Study Site and Organism

The herbivore exclusion and nutrient enrichment experiments were carried out at Al-Fahal reef about 13 km off the Saudi Arabian coast in the Central Red Sea (N22°18′19.98″, E38°57′46.08″; Figure S1) over a period of 16 weeks from June to September 2011. *Acropora hemprichii* is a common reef building coral present in the Red Sea and the Western Indian Ocean [43], which inhabits upper reef slopes at this study site in the Red Sea. Al Fahal reef does not fall under any legislative protection or special designation as a marine/environmental protected area. No special permit is required for the inshore coastal, reef, and intertidal areas around Thuwal. The Saudi Coastguard Authority under the auspices of KAUST University issued sailing permits to the site that includes coral collection. *Acropora hemprichii* is listed as vulnerable on the ICUN Red List (http://www.iucnredlist.org/details/132981/0).

### Experimental Design and Sampling

**Figure 1:**
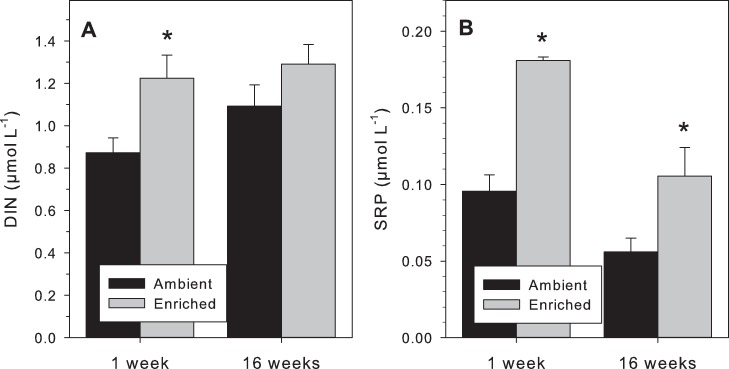
Inorganic nutrient concentrations. Shown are concentrations for ambient and enriched treatments of week 1 and week 16 directly above analyzed frames. Panel A depicts concentrations of dissolved inorganic nitrogen (DIN) and panel B the concentrations of soluble reactive phosphate (SRP). Asterisks indicate significance (*P<0.05*) of single t-tests.

The experimental setup included nine polyvinyl chloride (PVC) frames equipped with temperature loggers and stainless steel screws for coral finger attachment. Frames were deployed at 5–6 m water depth along a 40 m transect on a slightly sloped reef wall. Treatments were arranged in alternating order and separated from each other approximately by 2–5 m. Treatments were: (1) caging (CA), with a cage mesh size of 4 cm to simulate overfishing of larger herbivores; (2) fertilizer (FE), perforated PVC tubes filled with Osmocote fertilizer standard 15+9+12 (Scotts, Marysville, OH) embedded in 3% agarose to simulate nutrient enrichment; (3) a combination of caging & fertilizer (CF) (Figure S2). The fertilizer is composed of 15% nitrogen (in form of 7% nitrate and 8% ammonium), 9% phosphate (phosphorus pentoxide), and 12% potassium oxide. Furthermore, it contains magnesium oxide (2.5%), iron (0.45%), manganese (0.06%), copper (0.056%), zinc (0.020%), boron (0.020%), and molybdenum (0.025%). Fertilizer was deployed once without replenishments, but regular monitoring of inorganic nutrient concentrations assured continuous enrichment levels (Figure 1). Experimental treatments were conducted in triplicates, i.e. three *A. hemprichii* colonies (A, B, C) were collected from the same water depth in the area where the experiment was conducted. A total of nine frames were deployed (3 frames CA, 3 frames FE, 3 frames CF) each holding four coral fingers (for collection after 1 and 16 weeks) from each of the three mother colonies (A, B, C) yielding a total of 18 samples for physiological as well as for microbial analyses (3 treatments × 3 coral colonies × 2 time points). Experimental treatments started after attachment of coral fingers to the PVC frames via cable ties on stainless steel screws. After one and sixteen weeks, respectively, coral fingers were collected in sterile plastic bags and brought to the surface, where half of them were rinsed with filtered seawater (FSW) and subsequently shock-frozen in liquid nitrogen for microbial analyses and stored at −80°C until further processing. The other half of the coral fragments was used for incubations (see coral physiology section below). Water samples for microbial and nutrient analyses were collected with large ziplock bags directly above each frame prior to coral fragment collection during both sampling time points. These samples were transported on ice and 500 mL were subsequently filtered on 0.22 µm Millipore Durapore membrane filters for microbial analysis. Filters were stored at −80°C. Additionally, 50 mL seawater were filtered using 0.7 µm fiberglass filters (Whatman-GF/F) and stored at −20°C until inorganic nutrient analysis was carried out. The inorganic nutrients were photometrically analyzed by segmented debubbled continuous flow analysis (FlowSys - Alliance Instruments) according to [44] and for Ammonium according to [45]. Quality control was performed with a seawater certified reference material for nutrients (MOOS2 from NRC-CNRC).

### Coral Physiology

Incubation measurements were performed as follows: coral fingers were transferred to 1 L incubation glass jars in three opaque polyethylene (PE)-boxes filled with reef water (∼ 70 L) to keep samples at constant ambient temperatures during incubations. One jar per PE-box, filled only with reef water, served as a control. All PE-boxes were placed in the shade to avoid warming of the water. Dark incubation started after acclimatizing the samples for 1–2 hours in the dark PE-boxes. Initial O_2_ concentrations were measured with an O_2_ probe (HQ40d, Hach, Loveland, CO) inside each box after evenly distributing the water, with open incubation jars. The jars were then sealed and incubated over a period of 90–100 min before O_2_ concentrations in each jar were measured as described above. During incubations the boxes were carefully moved every 5 minutes to cause stirring of water inside the jars. Onset HOBO temperature loggers (Onset Computer Corporation, Pocasset, MA) in each box assured that water temperature during incubation remained constant (temperature differences between incubation jars and *in-situ* temperatures measured at PVC frames ranged between 0.5 and 1.6°C). All samples were subsequently stored on ice until further processing. Net O_2_ consumption was calculated subtracting end concentrations from the start concentrations. Normalization of data to µg O_2_ cm^−2^ h^−1^ was carried out by measuring the coral finger surface area using a cylinder as approximation according to the “simple geometry” model described by Naumann et al. [46] and by taking into account the exact incubation times. To investigate zooxanthellae abundance, chlorophyll *a* concentration, and δ^15^N isotopic signatures, tissue of each coral finger was washed with filtered sea water (FSW) before tissue was removed from the skeleton by air-blasting, collected in FSW, and homogenized using an Ultra-Turrax (T 18 basic, IKA, Staufen, Germany; 30 s at 3,500 rpm). Zooxanthellae abundance was counted using an Improved Neubauer hemocytometer (Hausser Scientific, Horsham, PA). Cell numbers were counted in 6 grid squares à 4 µm^3^ with 6 counts per sample (average count: 29 cells per grid square). To determine isotopic signatures of coral tissue and zooxanthellae, a modified version of [47] was used. The homogenate of coral tissue and zooxanthellae was centrifuged for 5 min in a tabletop centrifuge at 1,500 rpm to separate coral tissue and zooxanthellae. Recorded data were normalized to zooxanthellae cm^−2^ with surface area (see method described above). Homogenized coral tissue was first filtered on a GF/F filter and washed with MilliQ water to remove remaining salt. Subsequently, the filters were dried for 2–4 d at 40°C until constant weight and δ^15^N signatures were determined relative to atmospheric nitrogen in an isotope ratio mass spectrometer (Finnigan Corp., San Jose, CA). Isolated zooxanthellae were freeze-dried and directly measured with the mass spectrometer. For *Symbiodinium*-typing, DNA was extracted using the DNeasy Plant Mini Kit (Qiagen, Hilden, Germany) according to the manufacturer’s instructions. The ITS2 rDNA region was amplified with the primer pair ITSintfor2 and ITS2CLAMP [48] using PCR conditions described in [49]. Amplified fragments were separated on 8% polyacrylamide gels, following [50], and using a CIPHER DGGE KIT (CBS Scientific Company, Del Mar, CA). Gels were run at 150 V for 15 h and stained for 20 min with 1x SYBR Green (Invitrogen, Carlsbad, CA) and visualized on a Dark Reader Transilluminator (Clare Chemical Research, Dolores, CO). *Symbiodinium* types were determined by DGGE fingerprint profiles and sequencing. For sequencing, prominent band(s) were excised from the DGGE gel and re-amplified as described in [51]. Re-amplified products were purified following manufacturer’s instructions for Illustra ExoStar (GE Life Sciences, Piscataway, NJ). Samples were sent for bi-directional Sanger sequencing to the KAUST BioScience Core Laboratory (Thuwal, Saudi Arabia). Sequences were trimmed for quality in CodonCode Aligner (CodonCode Corporation, Centerville, MA). Forward and reverse sequences were assembled into contigs and aligned using ClustalW. Each contig was BLASTed against a local database of *Symbiodinium* ITS2 sequences. Coral respiration rates, chlorophyll *a*, zooxanthellae abundance, and δ^15^N isotopic signatures of corals and zooxanthellae were analyzed with Sigmaplot 12 (Systat Software, Point Richmond, CA) by two-way ANOVA. To meet parametric assumptions, δ^15^N coral data for colony-time factorial analysis were exponential transformed, and chlorophyll *a* and respiration data for genotype-time factorial analysis were square transformed.

### Coral Bacterial Community

DNA from flash frozen coral fragments and water filters was extracted using the Qiagen DNeasy Plant Mini Kit (Qiagen, Hilden, Germany) according to the manufacturer’s instruction. Coral tissue was separated from skeleton using high-pressure air and extraction buffer, while filters were bead-beaten with extraction buffer for 1 minute. For water samples, DNA of all filters from week 1 and of all filters from week 16 were pooled resulting in two water samples that represent a comprehensive reef water microbial composition from week 1 and week 16. For PCR amplification of the 16S rRNA gene, we used the primers 784F and 1061R that amplify *E. coli* position 781 to 1,060 [52]. The primer sequences were 5′CTATGCGCCTTGCCAGCCCGCTCAGtaAGGATTAGATACCCTGGTA3′ (784F) and 5′CGTATCGCCTCCCTCGCGCCATCAG(N)_8_ctCRRCACGAGCTGACGAC3′ (1061R). Primers include 454 LibA library adapters (underlined), a barcode (shown as N) [53], and a two base pair linker sequence to avoid barcode influence on the amplification (lowercase). PCRs were run in 30 µL triplicates per sample with Qiagen Multiplex PCR Kit (Qiagen, Hilden, Germany) and 30 ng/µL of input DNA using the following protocol: 15 min at 95°C, followed by 27 cycles of 95°C for 40 s, 55°C for 40 s, 72°C for 40 s, and a final extension cycle of 10 min at 72°C. PCR products were run on an 1% agarose gel to visualize successful amplification. Sample triplicates were pooled and then purified using PALL multi-well filter plates (Pall Corporation, Port Washington, NY) and a Millipore multiscreen HTS vacuum manifold (Millipore Corporation, Billerica, MA). DNA concentrations were measured using a Qubit 2.0 (Invitrogen, Carlsbad, USA) and adjusted to 30 ng/µL before subsequent pooling. The pooled sample ran on a 1% agarose gel to remove excess primers. DNA was subsequently isolated from the gel using the Qiagen MinElute Gel Extraction Kit (Qiagen, Hilden, Germany) according to manufacturer’s instructions. PCR products were sequenced using Titanium FLX chemistry on a quarter of a picotiter plate. Raw pyrosequencing reads were processed using the open source software mothur v.1.28.0 [54] for error correction, taxonomical classification using the greengenes database [55], and calculation of alpha-diversity and beta-diversity indices. More specifically, sequencing resulted in a total of 166,741 reads with a median length of 303 bp. The reads were split according to barcodes, error corrected, and quality trimmed using PyroNoise [56] as implemented in mothur, and subsequently aligned to the SILVA database alignment v102 [57]. We removed any sequences that did not cover positions 26,988 to 34,113 (variable regions 5 and 6 of the 16S rRNA gene). To reduce sequencing noise, a pre-clustering step as implemented in mothur (maximal two base pairs difference) was performed [58]. Further reads were removed after a check for chimeric sequences using UCHIME as implemented in mothur [59] and/or their identification as chloroplast or mitochondrial contamination. The resulting dataset of 112,414 sequence reads was used for all analyses. The mothur “classify.seqs” function was used to classify all sequences against the 2011 Green Genes database [55] as provided on the mothur webpage. For classification a bootstrap cutoff of 60% was used. For the UniFrac [60] analysis, the sequences were subsampled to the lowest number of sequences in any group (3,283 sequences, week 1, sample C, treatment CA). The Principal Coordinate (PCoA) and ANOSIM analysis were also performed in mothur, the plot was generated using the ggplot2 package [61] in R [62]. All sequences are available in the NCBI Sequence Read Archive (http://www.ncbi.nlm.nih.gov/sra) under accession number SRA062645. Mann-Whitney U test was conducted in Sigmaplot 12 (Systat Software, Point Richmond, CA). To determine distinct Operational Taxonomic Units (OTUs) that were significantly associated with *A. hemprichii* under treatments of overfishing, eutrophication, or both, we used the statistical package indicspecies [63]. We chose a conservative approach considering only OTUs that were highly significantly (*P*<0.01) associated with one or several sample groups.

## Results

### Environmental Parameters and Coral Physiology

Nutrient enrichment led to significant increases in dissolved inorganic nitrogen (DIN) and in soluble reactive phosphate (SRP) (Figure 1). A parallel study showed intensive algae growth (specifically filamentous algae) in caged and combined treatments on terracotta tiles that were installed on PVC frames identical to the ones used in this study [64]. As opposed to the tiles, the corals that were analyzed in this study were all free of algal overgrowth (with the exception of the sample from week 16, colony A, CF that died) during the study time, and we did not find significant differences between treatments, time, or a combination thereof for any of the investigated physiological coral parameters (Table 1, Figure S3). However, we did find significant differences between the coral colonies independent of *in-situ* treatments and those represented biological variance (Table 1, Figure S3). More specifically, coral samples from colony B differed from C in regard to respiration rates, colony A and B differed significantly from colony C in the chlorophyll *a* measurements and colony B differed from C in zooxanthellae abundance after week 16 (Figure S3). All coral specimens were associated with the same *Symbiodinium* clade over the duration of the experiment (clade C41), banding profile designation and sequence analyses were congruent (Figure S4). Hence, the differences observed cannot be attributed to differences of *Symbiodinium* type performance and origin of coral colony was more important than treatment effect.

**Table 1:** Results from 2-factorial ANOVA of physiological coral parameters.

		Coral respiration		Chlorophyll *a*		Zooxanthellae		δ^15^N Coral		δ^15^N Zooxanthellae	
	df	*F*	*P*	*F*	*P*	*F*	*P*	*F*	*P*	*F*	*P*
**Treatment vs. Time**											
Tr	2	0.146	0.866	0.439	0.655	0.380	0.693	0.419	0.668	0.495	0.622
Ti	1	0.198	0.666	2.635	0.133	4.328	0.062	3.468	0.089	2.335	0.155
Tr × Ti	2	0.882	0.444	0.218	0.807	0.471	0.636	1.468	0.272	1.425	0.282
**Colony vs. Time**											
C	2	4.814	0.034*	11.617	0.002*	2.653	0.115	0.266	0.771	1.918	0.193
Ti	1	0.005	0.947	6.563	0.026*	9.071	0.012*	3.393	0.093	3.931	0.073
C × Ti	2	2.284	0.152	2.671	0.113	3.599	0.063	0.611	0.560	1.240	0.327

Response variables are shown in the first row and the independent factors in the first column.

Abbreviations: Tr = Treatment, Ti = Time, C = Coral Colony. Significant results (*P*<0.05) are indicated by asterisks.

### Microbial Community of Corals and Reef Water

We sequenced a total of 166,741 reads, of which 112,414 were left after error correcting, chimera detection, and undesirable mitochondrial and chloroplast sequence removal. We retrieved the highest number of reads from the water sample from week 1 (21,155 sequence reads), and the lowest number of reads from coral colony C, week 1, cage treatment (3,283 sequence reads). One coral fragment was dead after 16 weeks and left out from the remainder of the analyses (colony A, cage & fertilizer treatment). Good’s estimator of coverage [65] showed that the majority of bacterial diversity was captured in the sequence data with values ranging from 0.90 to 0.98 (Table 2). The number of bacterial operational taxonomic units (OTUs) in corals varied from 104 (coral C, week 1, cage & fertilizer treatment) to 908 (coral B, week 16, treatment FE) spanning almost an order of magnitude difference in diversity between samples. We found a significantly higher number of OTUs in all coral samples from week 16 in comparison to week 1, irrespective of treatment and despite a higher average number of reads in samples from week 1 (MWU, *P* = 0.011, median number of OTUs week 1 = 120, median number of OTUs week 16 = 432, all specimens were subsampled to 3,283 reads). This observation is also supported when counting the number of distinct OTUs that we detected. We identified a total of 4,442 distinct OTUs of which 3,290 were found in corals and 1,432 in water. After week 1 we found 818 OTUs in all coral samples, after week 16 we identified 2,780 OTUs in all coral samples. Accordingly, bacterial diversity of corals increased over time. The Inverse Simpson Index for diversity varied from 2.14 (coral C, week 1, cage & fertilizer treatment) to 16.41 (coral A, week 16, fertilizer treatment). Overall, bacterial diversity between corals was highly variable but lower than in the water column. However, Simpson Evenness estimates ranged from 0.01 to 0.04 indicating few dominant bacterial taxa.

**Table 2:** Summary statistics of 454 16S rRNA gene sequencing (CA = cage, FE = fertilizer, CF = cage & fertilizer).

Time	Coral Colony	Treatment	# of Sequences	Sequence Coverage	Number of OTUs	Inverse Simpson Index	Chao1	Simpson Evenness
Week 1	A	CA	4882	0.98	184	2.51	398.24	0.01
	A	FE	6175	0.98	157	2.30	417.00	0.01
	A	CF	4368	0.98	177	2.50	373.20	0.01
	B	CA	7167	0.98	206	5.54	432.11	0.03
	B	FE	3938	0.98	149	5.77	457.08	0.04
	B	CF	6595	0.98	143	2.21	353.91	0.02
	C	CA	3283	0.96	175	2.19	774.44	0.01
	C	FE	5489	0.99	139	5.26	353.50	0.04
	C	CF	4423	0.98	104	2.14	286.57	0.02
	Water	–	21155	0.97	919	25.01	2947.22	0.03
Week 16	A	CA	5331	0.96	411	5.27	922.88	0.01
	A	FE	4158	0.91	659	16.41	1304.32	0.02
	A	CF	–	–	–	–	–	–
	B	CA	5339	0.90	888	8.06	1764.99	0.01
	B	FE	7505	0.92	908	5.72	2360.50	0.01
	B	CF	3595	0.86	761	8.22	1880.81	0.01
	C	CA	3592	0.92	364	2.78	1160.38	0.01
	C	FE	5086	0.96	336	3.00	786.19	0.01
	C	CF	3965	0.98	112	2.20	373.77	0.02
	Water	–	6368	0.92	724	23.50	2929.85	0.03

Sample Week 16, colony A, CF died and was left out of the analysis.

**Figure 2:**
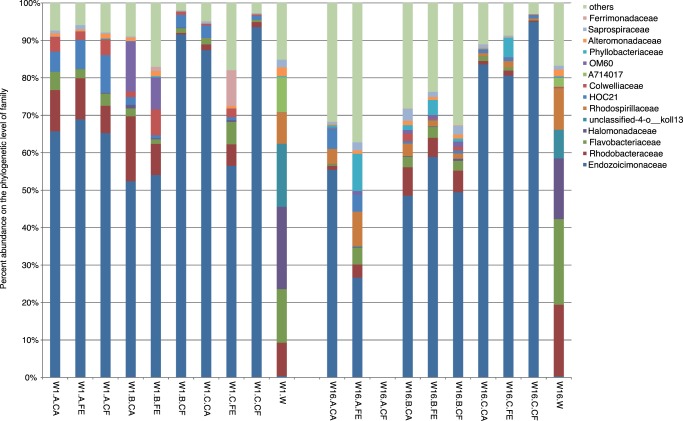
Bacterial taxonomy stack plot on the phylogenetic level of family. Each color represents each of the 15 most abundant families in all samples. All other taxa are grouped under category others. Bars are plotted according to 1) time (W1 =  week 1, W16 =  week 16), 2) coral mother colony (A, B, C) or water (W), and 3) treatment (CA = cage, FE = fertilizer, CF = cage & fertilizer). Sample W16.A.CF died and was left out of the analysis.

We classified all sequences to the family level in order to look into the composition of the sample-specific microbial assemblages (Figure 2). Water samples were markedly different from coral samples, but similar to each other. Bacteria of the family Endozoicimonaceae in the order Oceanospirillales of the class Gammaproteobacteria dominated the coral microbial community. Depending on the coral sample, bacteria of the family Endozoicimonaceae made up between 27% and 95% of the microbial community of *A. hemprichii*, irrespective of time point or treatment. Most interestingly, only two OTUs made up>99% of all *Endozoicomonas* bacteria (these OTUs could not be classified to the species level). In total, seven OTUs (Otu0001, Otu0002, Otu0003, Otu0011, Otu0029, Otu0055, Otu0085) were consistently found across all coral samples and on average accounted for >72% of the total number of bacteria (Table S1). Of these seven OTUs, the first two were classified as *Endozoicomonas* sp. and made up 69% of all reads. The remaining five OTUs consisted of three Proteobacteria (families Rhodobacteraceae, Burkholderiaceae, Hyphomonadaceae) and two bacteria in the phylum Bacteroidetes (family Flavobacteriaceae) that made up 3% of all reads. Hence, in contrast to the substantial microbial richness identified in *A. hemprichii* (Table 2), the majority of bacteria in the coral samples were covered by only few bacterial species.

**Figure 3:**
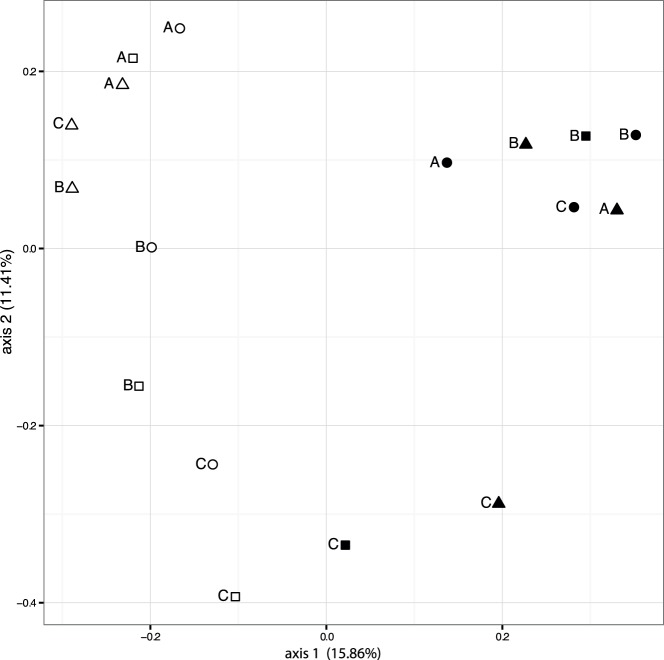
Principal Coordinate Analysis (PCoA) of unweighted UniFrac distances between coral samples. White symbols represent samples from week 1. Black symbols are samples from week 16. Coral colonies are depicted by the letter preceding the symbol (A, B, C). Circles denote cage, triangles denote fertilizer, and squares denote cage & fertilizer treatments. Percentages given represent the amount of variance explained by the corresponding axis.

To further analyze differences in bacterial community composition between colonies, treatments, and time points (beta diversity), we summarized unweighted UniFrac distances between samples with principal coordinate analysis (PCoA) [60] (Figure 3). This analysis clustered the microbial communities according to time (ANOSIM *P*<0.001), and also, but not significantly, according to coral colony and/or treatment (e.g. colony A, week 1). Interestingly, specimens from week 1 separated mainly along axis 2, whereas specimens from week 16 separated mainly along axis 1, indicating that the factor(s) driving community composition were different for both time points.

### Bacterial Species Associated with Nutrient Enrichment and Herbivore Exclusion in Corals

In addition to community level patterns, we analyzed the association of single OTUs to time points and experimental treatments. We identified ten OTUs that were significantly associated to one or more experimental treatments at a significance level of *P*<0.01 (Table 3). One OTU (genus *Nautella*, species unclassified) was significantly associated with the cage & fertilizer treatment in week 1, but was also present in the cage & fertilizer treatment in week 16 and the cage treatment in week 1. After week 16, we identified nine OTUs that were significantly associated with an experimental treatment or a combination thereof. Two OTUs were overrepresented upon cage and fertilizer treatments, but interestingly not in the combined cage & fertilizer treatment. Note that samples from only two colonies were analyzed for the combined treatment in week 16. The two OTUs belonged to the phylum Proteobacteria in the families Desulfovibrionaceae (*Desulfovibrio capillatus*) and NB1-i (not further classified) and were not identified in week 1. Seven OTUs were significantly enriched upon fertilizer exposure. Of these OTUs, a bacterium from the genus *Defluvibacter* showed a very strong increase in abundance in comparison to all other treatments and time points. OTU counts ranged from 111 to 285 in all three coral specimens in week 16. In comparison, in all other treatments this OTU was either absent or present in low numbers (week 1, FE, 1 to 10 OTUs). The remaining six OTUs were exclusively identified after week 16 and belonged to the families Balneolaceae, Marinilabiaceae, and Saprospiraceae (phylum Bacteroidetes) and Phyllobacteriaceae, Rhodobacteraceae, Campylobacteraceae (phylum Proteobacteria).

**Table 3:** Bacterial OTUs and non-subsampled read counts significantly associated with experimental groupings.

	Week 1										Week 16								
	Cage				Fertilizer			Cage & Fertilizer			Cage			Fertilizer			Cage & Fertilizer		
	A		B	C	A	B	C	A	B	C	A	B	C	A	B	C	A	B	C
OTU0203	1		1	0	0	0	0	**3**	**3**	**2**	0	0	0	0	0	0	–	1	0
OTU0223	0		0	0	0	0	0	0	0	0	**5**	**5**	**1**	**4**	**4**	**8**	–	0	2
OTU0785	0		0	0	0	0	0	0	0	0	**1**	**2**	**1**	**1**	**2**	**1**	–	0	0
OTU0019	0		0	0	2	10	2	0	0	0	0	0	0	**365**	**226**	**254**	–	0	0
OTU0130	0		0	0	0	0	0	0	0	0	0	1	0	**9**	**14**	**7**	–	1	0
OTU0515	0		0	0	0	0	0	0	0	0	0	1	0	**2**	**7**	**3**	–	0	0
OTU0835	0		0	0	0	0	0	0	0	0	1	0	0	**1**	**2**	**1**	–	0	0
OTU1390	0		0	0	0	0	0	0	0	0	0	0	0	**1**	**1**	**1**	–	0	0
OTU0672	0		0	0	0	0	0	0	0	0	1	1	0	**2**	**2**	**3**	–	0	0
Taxonomic assignment of OTU (bootstrap value)																			
OTU0203		p__Proteobacteria(100);c__Alphaproteobacteria(100);o__Rhodobacterales(100);f__Rhodobacteraceae(100);g__Nautella(57);sp__unclassified(57);																	
OTU0223		p__Proteobacteria(100);c__Deltaproteobacteria(100);o__Desulfovibrionales(100);f__Desulfovibrionaceae(77);g__Desulfovibrio(77);s__Desulfovibriocapillatu(77);																	
OTU0785		p__Proteobacteria(100);c__Deltaproteobacteria(100);o__NB1-j(100);f__NB1-i(100);g__unclassified(100);s__unclassified(100);																	
OTU0019		p__Proteobacteria(100);c__Alphaproteobacteria(100);o__Rhizobiales(100);f__Phyllobacteriaceae(100);g__Defluvibacter(100);s__unclassified(100);																	
OTU0130		p__Bacteroidetes(100);c__Sphingobacteria(100);o__Sphingobacteriales(100);f__Balneolaceae(100);g__Balneola(100);s__Balneolaalkaliphil(100);																	
OTU0515		p__Bacteroidetes(100);c__Bacteroidia(100);o__Bacteroidales(100);f__Marinilabiaceae(100);g__unclassified(100);s__unclassified(100);																	
OTU0835		p__Proteobacteria(100);c__Alphaproteobacteria(100);o__Rhizobiales(100);f__Phyllobacteriaceae(100);g__unclassified(100);s__unclassified(100);																	
OTU1390		p__Bacteroidetes(100);c__Sphingobacteria(100);o__Sphingobacteriales(100);f__Saprospiraceae(100);g__unclassified(75);s__unclassified(75);																	
OTU0672		p__Proteobacteria(100);c__Alphaproteobacteria(100);o__Rhodobacterales(90);f__Rhodobacteraceae(90);g__Rhodobaca(90);s__unclassified(90);																	
OTU0706		p__Proteobacteria(100);c__Epsilonproteobacteria(100);o__Campylobacterales(100);f__Campylobacteraceae(100);g__Sulfurospirillum(67);s__unclassified(67);																	

All identified OTUs were identified with a significance level of *P*<0.01. Bold numbers designate the time points and treatments of significant association.

## Discussion

### Environmental Parameters

With a complex experimental design that entails single and combined treatments, we were aiming at better separating the relative contribution of nutrient enrichment and overfishing to a combined manipulation. Interestingly, neither coral physiology data nor bacterial community data indicated that the combined treatment of cage & fertilizer is a mere combination of the effects of each treatment on its own. Rather, each treatment produced a distinct and complex response that was not necessarily identified in the simultaneous and combined treatment. Furthermore, as a consequence of conducting a time course study during the summer months, all samples were subject to an increase and subsequent decrease in water temperature over the course of the project. While the average water temperature of week 1 was 28.7°C (min: 28.1°C, max: 29.3°C), the average water temperature after 10 weeks was 31.8°C (min: 31.2, max: 32.3), and the average water temperature in week 16 was 30.2°C (min: 28.9°C, max: 30.9°C). We see that the microbial community increases over time in all coral samples and this could be attributable to the variable temperature regime all samples were exposed to. Secondly, the coral fingers where subjected to the experimental conditions after being harvested from the respective mother colony without a time of acclimation. However, coral holobiont performance did not show a dependence on time, as physiological measures were similar after week 1 and week 16. Rather, physiology had a strong association with the coral colony the samples were taken from. Furthermore, all coral fingers were sampled at the same time, so that differences between treatments did still reflect biological variation as a response to experimental treatment.

### Coral Physiology

A study by [66] found that turf algae reduce the effective photochemical efficiency of neighboring corals. Data from an experiment that was conducted in parallel showed that algal biomass had significantly increased over time as a result of reduced herbivory and in the combined treatment of reduced herbivory and increased nutrients, but not as a result of nutrient enrichment alone [64]. Here, we did not find significant differences between the treatments in coral holobiont performance as measured by respiration rates, chlorophyll *a* levels, zooxanthellae abundance, or coral and algal isotopic nitrogen ratios. Hence, the consequences of overfishing and eutrophication on coral holobiont physiology in this study may have been too subtle to affect measured parameters. At the same time, however, high nutrient loads do not necessarily have negative effects on holobiont performance (e.g. photosynthetic performance) as long as they are balanced [11]. In this study, we used a commercially available fertilizer that was composed of a balanced nutrient composition. In the water column, however, we found a stronger relative increase in SRP concentrations between ambient and enriched treatments than for DIN (Figure 1). Alternatively and/or additionally, the coral *Acropora hemprichii* may have proven more resilient to our experimental treatments than other coral species that have not been investigated here. Last, our experiment lasted for a total of 16 weeks. Overfishing and nutrient enrichment are often anthropogenically induced processes that can last much longer than the here chosen time frame, so that we may also have to consider a temporal component. Nonetheless, we were able to detect differences in the microbial community structure upon treatments and this underlines the importance of conducting field studies in a holobiont framework, taking both physiological and molecular measures into account.

### Microbial Community of Corals and Reef Water

While we did not see significant differences in holobiont physiology as a result of either of the treatments, we could clearly pinpoint differences in the microbial community after treatments and in both time points. Most notably, the majority of bacteria (>72%) did not change over time or treatment and were comprised of only seven OTUs. Accordingly, microbial changes were proportionally small and overall holobiont function (as indicated by the physiological data) may have been ‘conserved’. Nonetheless, our data indicate that *A. hemprichii* harbors hundreds of different OTUs and our results are in accordance with previous estimates of coral microbial diversity [31], [33]. In total, 4,442 distinct OTUs were detected in water and coral samples, 3,290 of which were associated with coral specimens. This number was highly variable though in regard to time point, treatment, and even coral colony. However, we saw a general increase in bacterial diversity after 16 weeks in comparison to week 1. The latter indicates that the complexity of microbial assemblage is more a function of environment than mother colony. In contrast, bacterial diversity in water was stable over time. We conclude that the general increase of microbial complexity is specific to the coral and does not come from an increase in diversity over time in the surrounding water column. Several other studies [32], [67] found a stable microbial community over time, however, coral-associated bacterial communities analyzed by [35] were not stable and grouped according to sampling time.

*Endozoicomonas* have now been identified in a number of studies and seem to be present in many marine invertebrates [33], [68]–[74]. While we report on samples from a limited geographic range, we assume that *Endozoicomonas* is predominant in *A. hemprichii* across its range, given its strong presence irrespective of time or treatment in our study. Furthermore, it is interesting to note that only two OTUs made up>99% of all *Endozoicomonas* bacteria. This indicates that the association of *Endozoicomonas* to *A. hemprichii* is very specific. Different members of the Oceanospirillales order (to which *Endozoicomonas* belong) include obligatory heterotrophic rod-like bacteria known for biofilm production that allows other bacteria to colonize surfaces [73]. It is tempting to speculate on such a function for *Endozoicomonas* suggesting that they may play a key symbiotic role as an “architect microbe” that structure and contribute to microbial community function.

UniFrac distance significantly separated samples according to time. However, it seems that the factors driving sample separation were not the same between both time points as samples from week 1 show little differentiation on axis 1 but high differentiation on axis 2. In contrast, and to a lesser degree, samples from week 16 did separate along axis 1 and showed less variance on axis 2. In line with a longer treatment exposure we would expect a stronger separation by treatment after week 16, but the phenotypic reaction may be complex and the clustering of samples from week 16 a result of origin, treatment effect, and time. The results from our microbial species analysis however do support a stronger treatment effect with time.

### Bacterial Species Associated with Nutrient Enrichment and Herbivore Exclusion in Corals

In the analysis of bacterial OTUs specifically associated with treatments, we found more OTUs in single treatments after week 16 (nine OTUs vs. one OTU after week 1), and we also found a higher abundance of significantly associated OTUs after week 16. The finding that coral associated microbial communities respond to treatments supports previous findings that show that bacterial assemblages vary under different conditions such as increased temperature, elevated nutrients, dissolved organic carbon loading, and reduced pH [13]. Interestingly, the combined cage & fertilizer treatment did not show the largest effect size. At the same time, however, the sample for microbial community analysis from colony A of the combined cage & fertilizer treatment died in the process of the experiment. As a consequence, we had less statistical power to detect significant associations of OTUs to the treatment effect. Accordingly, the only significantly associated OTU we identified after week 1 was found in the combined cage & fertilizer treatment. This OTU belongs to the genus *Nautella* representing marine bacteria belonging to the Roseobacter lineage of the Alphaproteobacteria. Up to date little is known about this type of bacteria but one member, *Nautella* sp. R11, has been shown to cause bleaching in the temperate-marine macroalga *Delisea pulchra* while switching from an opportunistic to a pathogenic lifestyle [75]. Overall, fertilizer treatment resulted in more associated OTUs than caging, and the two OTUs that were identified in caging after week 16 were also found in the fertilizer treatment after week 16 (Table 3). These two OTUs belonged to the phylum Proteobacteria, families Desulfovibrionaceae and NB1-i. While there is insufficient data available on bacterial species belonging to NB1-i, some members of the family Desulfovibrionaceae are sulfate-reducing [76]. We identified seven OTUs enriched in the fertilizer treatment after week 16 that were not enriched in any other treatment or time point. This notion points towards specific selection regimes under eutrophication, rather than a ‘common’ microbial community response as a result of general environmental disturbance. A bacterium from the genus *Defluvibacter* was highly abundant. *Defluvibacter* are aerobic denitrifiers, and members of this family are found in activated sludge from wastewater treatment plants [77], [78]. It is also interesting to note that this bacterium is aerobic in contrast to the sulfate-reducing bacteria common in hypoxic environments that we identified after week 1 as a result of a combined treatment of nutrient-enrichment and caging. This indicates again that distinct bacterial species may gain a selection advantage and increase in abundance as a consequence of a specific treatment, rather than non-selective growth of opportunistic bacteria regardless of the underlying treatment. Of the other bacteria significantly abundant after 16 weeks in the fertilizer treatment, we identified members of Marinilabiaceae and Saprospiraceae. Bacteria of these families are found in nutrient rich environments such as whale fall or domestic and industrial wastewater treatment plants [79]–[81]. Last, we identified members of the Rhodobacteraceae that comprise a very diverse group with heterotrophic and phototrophic members [82], so that any functional implication is difficult. However, Rhodobacteraceae appear to do well under conditions of environmental change, i.e. many members seem to arise opportunistically [83]–[85]. Taken together, we were only able to derive species level annotations for two of the ten OTUs significantly enriched as a function of experimental treatment. Nevertheless, the functional associations we derived from the phylogenetic assignments correspond to the anticipated environmental consequences of a given treatment. For instance, we found bacteria from the family of Phyllobacteriaceae that contains denitrifying and nitrogen fixing bacterial species in the nutrient-enriched fertilizer treatment regime. Some bacteria within this family may probably be benefitting from the provided nitrogen (denitrifyers), others may be utilizing the iron and molybdenum of the fertilizer, both latter are critical metal co-factors for nitrogenase [86], the enzyme responsible for nitrogen fixation. Nitrogen fixation is an energetically costly process. Accordingly, we would not expect nitrogen fixing bacterial species to be enriched if other N-sources such as provided by the fertilizer were available. Experimental work, however, demonstrates that N_2_ fixation still occurs at high ambient concentrations of nitrate [87]–[91]. Furthermore, N_2_ fixation in eutrophic environments may be attributed to protecting the enzyme nitrogenase from inactivation [92], [93].

### Conclusion

This study analyzed the distinct and combined effects of nutrient enrichment and herbivore exclusion via *in situ* fertilizer diffusion, caging, and both over a period of 16 weeks on a coral reef in the Central Red Sea. While the physiology of the coral holobiont did not show significant differences in regard to O_2_ consumption, zooxanthellae counts and identity, chlorophyll *a*, or nitrogen isotopic ratios in the coral tissue as a function of experimental treatment, the bacterial communities derived from the different treatments illustrate that the microbial assemblage of the coral holobiont is variable and a consequence of mother colony, environmental conditions, and time. This underlines the importance of conducting field studies in a holobiont framework, taking both, physiological and molecular measures into account. Secondly, the functional associations of overrepresented bacteria in the treatments corresponded well with the environmental footprints of a given treatment (e.g. we found nitrogen fixing and denitrifying bacterial species in the nutrient-enriched fertilizer treatment regime). However, they were not stable over time indicating that the presence of potential indicator bacterial species may vary. Notably, the majority of bacterial cells of *A. hemprichii* were provided by a few OTUs of the genus *Endozoicomonas* that formed a stable association.

## Supporting Information

Figure S1 **Study site.** Right panel shows position of the study area in the Red Sea. The circle on the left panel indicates study site at the Northern tip of Al Fahal-reef about 13 km off the Saudi-Arabian coast.
(EPS)

Figure S2 **Treatment scheme.** Coral fingers were attached to stainless steel screws on PVC frames with cable ties. A: cage treatment imitating overfishing pressures, frame with coral fragments underneath. B: fertilizer treatment imitating eutrophication pressures with slow releasing fertilizer diffusing out of the red bars. C: combined treatment of cage & fertilizer as in A and B.
(EPS)

Figure S3 Physiological parameters of corals and zooxanthellae. The left column shows the comparisons of treatments, and the right column the comparisons of coral colonies. Given P-values are from a 2-factorial ANOVA. See Table 1 for full results. Abbreviations used: Tr = Treatment, Ti = Time, G = Genotype. Asterisks indicate significant differences between two groups.
(EPS)

Figure S4 **DGGE banding pattern of ITS2 from all coral samples.** Please note that the coral samples are not depicted in a temporal order.
(PDF)

Table S1 **Overview over sequence counts, taxonomic classification, and 16S reference amplicon sequence for all OTUs identified.**
(XLSX)
