# Correction: *In-situ* Effects of Eutrophication and Overfishing on Physiology and Bacterial Diversity of the Red Sea Coral *Acropora hemprichii*

**DOI:** 10.1371/annotation/be4a3168-5284-4083-b5ed-5cd0f4630823

**Published:** 2013-11-14

**Authors:** Christian Jessen, Javier Felipe Villa Lizcano, Till Bayer, Cornelia Roder, Manuel Aranda, Christian Wild, Christian R Voolstra

The last row of Table 3 is missing. Please find the complete table 3 here: 

**Figure:**
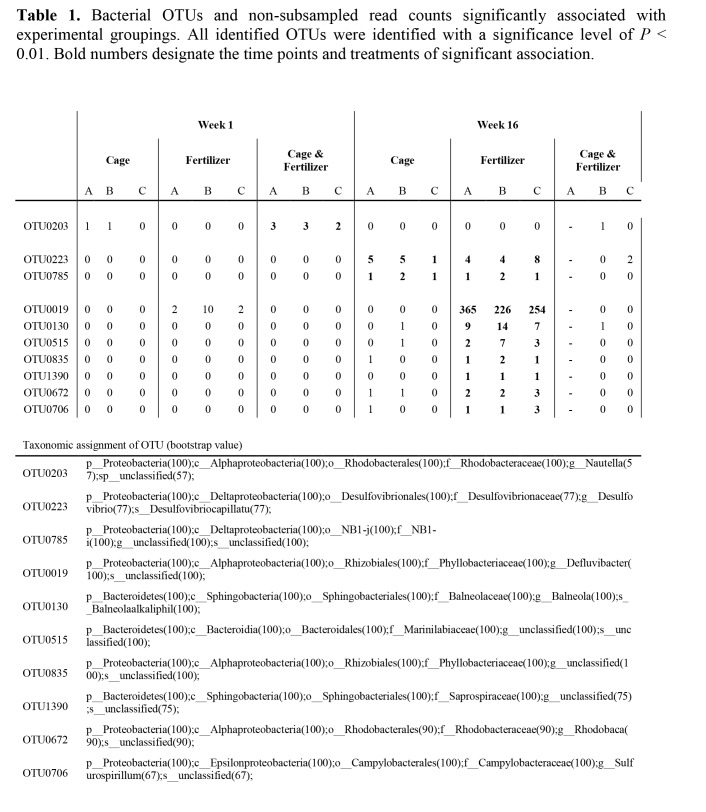


In the body of the text under the section "Bacterial Species Associated with Nutrient Enrichment and Herbivore Exclusion in Corals," the second to last sentence should read: Read counts ranged from 226 to 365 in all three coral specimens in week 16. In comparison, in all other treatments this OTU was either absent or present in low numbers (week 1, FE, 2 to 10 reads).

In the body of the text under the section "Bacterial Species Associated with Nutrient Enrichment and Herbivore Exclusion in Corals," the sentence, "It is also interesting to note that this bacterium is aerobic in contrast to the sulfate-reducing bacteria common in hypoxic environments that we identified after week 1 as a result of a combined treatment of nutrient-enrichment and caging." should be deleted. This sentence is referring to an OTU that was found after 16 weeks (OTU0223) and not after 1 week (OTU0203). 

