# Supplementary material for: In-situ Effects of Eutrophication and Overfishing on Physiology and Bacterial Diversity of the Red Sea Coral Acropora hemprichii
Source: PLoS One. 2013 Apr 22;8(4):e62091. doi: 10.1371/journal.pone.0062091 (PMC3632597; doi:10.1371/journal.pone.0062091)
Supplement: Figure S4 — DGGE banding pattern of ITS2 from all coral samples. Please note that the coral samples are not depicted in a temporal order. (PDF) [file pone.0062091.s004.pdf]

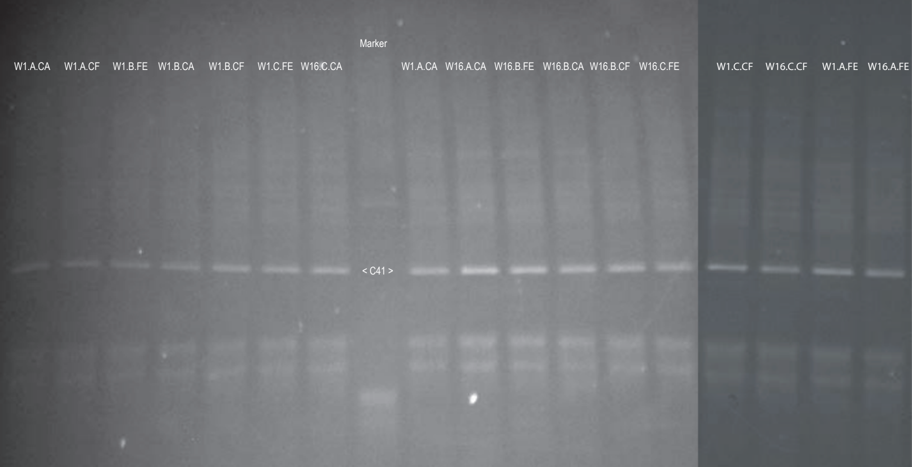
Marker
W1.A.CA
W1.A.CF
W1.B.FE
W1.B.CA
W1.B.CF
W1.C.FE
W16.C.CA
W1.A.CA
W16.A.CA
W16.B.FE
W16.B.CA
W16.B.CF
W16.C.FE
W1.C.CF
W16.C.CF
W1.A.FE
W16.A.FE
< C41 >
